# Influence of Cryogenic Cyclic Aging on Room-Temperature Mechanical and Tribological Performance of Polyimide-Based Materials

**DOI:** 10.3390/polym18131651

**Published:** 2026-07-02

**Authors:** Maksim Nikonovich, Amilcar Ramalho, Nazanin Emami

**Affiliations:** 1 Polymer-Tribology Group, Division of Machine Element, Luleå University of Technology, 971 87 Luleå, Sweden; 2 Department of Mechanical Engineering, University of Coimbra, 3030-788 Coimbra, Portugal; amilcar.ramalho@dem.uc.pt

**Keywords:** polyimide, tribology, cryogenic cyclic aging, vacuum, composite

## Abstract

Cryogenic environments impose severe thermal and mechanical stresses on polymer components, yet the effects of long-term cryogenic cycling on their subsequent room-temperature performance remain insufficiently understood. This study investigated the influence of cryogenic cyclic aging on the mechanical and tribological behaviour of polyimide (PI)-based materials, including neat PI and composites reinforced with MoS_2_, graphite, and/or PTFE. Repeated cryogenic cycling was followed by mechanical characterisation and tribological testing at 25 °C in air and vacuum. This work systematically compares neat and filled PI materials after cryogenic cyclic aging and correlates mechanical changes with transfer-film formation and wear behaviour. Cryogenic cyclic aging had only minor effects on weight and thermal stability but significantly altered the viscoelastic behaviour, increasing creep and residual strain, with variations depending on the polymer structure and filler content. Fracture toughness showed a statistically significant improvement only for PI2 (up to 93%). Changes in PI1, PI3, PI4, and PI5 fell within the experimental scatter and were interpreted as non-significant trends. In air, abrasive wear dominated in unreinforced PI, while graphite/PI composites exhibited adhesive wear and improved transfer film formation, reducing wear rates by up to 26%. In vacuum, the wear rate of aged graphite/PI increased by up to two orders of magnitude.

## 1. Introduction

Nowadays, many components in the energy, aerospace, transportation, and superconducting industries are subjected to a variety of harsh conditions. They often include cryogenic temperatures, high-pressure gradients, rapid temperature changes, and chemical and physical interaction with the environment, often applied simultaneously. Such conditions can reduce the durability and safety of a system and raise its operating and maintenance costs. Advances in technology have created a need for materials that can withstand these extremes. Due to strict application requirements, traditional materials cannot be directly used in new cryogenic systems without comprehensive research and testing.

The inability to apply external lubrication at low temperatures and in vacuum has led to the use of various self-lubricating polymers such as polyetheretherketone (PEEK), polytetrafluoroethylene (PTFE), polyamide, and polyimide (PI). Polyimides possess high thermal stability and good mechanical properties and maintain stable tribological performance across a broad temperature range [1,2,3]. The mechanical and tribological performance of polyimides can be altered or tailored to fit special requirements. Changes in polymer structure may lead to improvements in mechanical properties [4,5,6,7,8,9,10,11]. Neat polyimide is known to form a thin and oriented transfer film on a countersurface via a reaction [2,12]. However, research shows that polyimides are sensitive to water content, which can affect tribological properties by inhibiting the formation of transfer films, thus increasing friction and wear in humid conditions [1,2,13]. The addition of solid lubricants can enhance performance by forming a low-friction transfer film on the countersurface, reducing wear rates [13]. However, reinforcement can also compromise the mechanical strength due to the presence of filler/matrix interfaces, where variations in temperature and load might induce stress accumulation [14,15]. Fillers like MoS_2_ and PTFE, due to their layered or band structures, respectively, have demonstrated effectiveness in reducing friction in vacuum environments by creating durable transfer films [16,17]. Graphite has shown a limited impact on friction across different environments due to carbon bond saturation or carbon dusting [13,18].

Cryogenic temperatures are known to improve the mechanical and tribological performance of many neat polymers due to physical shrinkage and restricting molecular movement [19,20]. In particular, the Young’s modulus, tensile strength, and hardness increase as the temperature falls, often at the cost of embrittlement caused by a lower elongation at break [14,21,22]. Although the tribological performance of polyimides generally improves in cryogenic environments [9,23,24,25,26], some studies report a higher coefficient of friction (CoF) and wear rate due to a change in wear mechanism [16,25].

Exposure to cryogenic temperatures, especially under a gradient of temperature or load, can cause the physical and/or chemical aging of polymers and change their mechanical and tribological performance. Most frequently, the effects of thermal aging are studied at elevated temperatures. Ragosta et al. [10] and Lin et al. [27] found structural changes in polyimide after high-temperature aging, although the mechanical properties were not significantly affected. Khazaka et al. [28] revealed the influence of the aging environment, reporting limited thermo-oxidative degradation in air and nitrogen.

Cryogenic aging has been studied only occasionally. Many papers consider only short exposures, often limited to a few hundred hours, and their effects on mechanical properties. Dasari et al. [29,30] suggested that short-term cryogenic aging (up to 16 h) can relieve thermal stresses and may improve flexural properties, while longer aging gave mixed results [31,32,33]. According to Najafi et al. [34], incorporating glass fibres and nano-clay into epoxy matrices led to diminished flexural strength. In contrast, Kim et al. [35] observed that exposing graphite/epoxy composites to cryogenic conditions at −150 °C for 30 min did not noticeably affect their mechanical behaviour. In previous studies [36,37], the authors examined the effects of long-term cryogenic static aging in liquid nitrogen lasting 5 months on PEEK and PI composites, and they found a reduction in fracture toughness and wear resistance for some compositions.

More recently, Xue et al. [38] reported that cryogenically aged PTFE exhibited a three-stage room-temperature creep response, where moderate aging improved its creep resistance but excessive aging deteriorated its performance. Liu et al. [39] showed that PTFE gaskets subjected to cryogenic liquid-oxygen immersion aging exhibited aging- and stress-dependent fatigue–creep behaviour. Torskaya et al. [40] found that climatic aging reduced the friction coefficient and improved the wear resistance of rubber–UHMWPE composites after two months, whereas a higher temperature increased wear.

Although the short-term cryogenic aging of polymers has been studied several times, work on the long-term effects on mechanical properties is still scarce. To the best of the authors’ knowledge, the effect of prolonged cryogenic cyclic aging on the subsequent tribological performance of PI-based materials has not been studied systematically, particularly in both air and vacuum. The novelty of this work is therefore a combined assessment of the changes induced by cryogenic cyclic aging in the thermomechanical responses, filler/matrix interactions, transfer film formation, and wear mechanisms of neat PI and selected PI composites.

This study therefore aims to (1) assess how prolonged cryogenic cyclic aging affects the thermomechanical behaviour of PI-based composites and (2) evaluate the combined effects of cyclic aging and the test environment on the tribological performance of PI-based materials, including the wear mechanisms and the development of transfer films relative to non-aged materials.

## 2. Materials and Methods

### 2.1. Materials

Five commercially available materials based on amorphous thermoplastic polyimides (see Table 1 for more details) were investigated. The PI1–PI4 materials belonged to the same commercial family, featuring polyimides based on pyromellitic dianhydride (PMDA) and 4,4′-diaminodiphenyl ether (ODA) (structure 1), while the PI5 material was derived from a different monomer (structure 2).

### 2.2. Experimental Procedures

#### 2.2.1. Cryogenic Cyclic Aging Protocol

**Figure 1:**
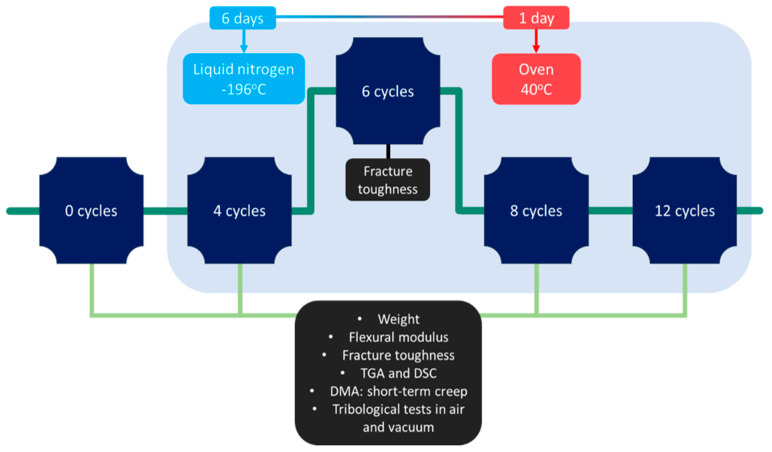
Experimental workflow showing the cryogenic cyclic aging protocol and characterisation sequence for PI-based materials.

Specimens underwent twelve cryogenic aging cycles. Each cycle consisted of 6 days of immersion in liquid nitrogen (−196 °C), followed by 24 h of heating in an oven at 40 °C (with relative humidity of 30%). Key material properties were assessed at intervals throughout the process after the completed aging cycle, including weight changes, thermal stability, mechanical behaviour, and tribological performance. The experimental workflow, including the cryogenic cyclic aging protocol and subsequent characterisation steps, is summarised in Figure 1.

#### 2.2.2. Weight Characterisation

Specimen weights (initially ranging between 550 and 600 mg) were measured using a Mettler Toledo AX205 balance (Mettler Toledo GmbH, Greifensee, Switzerland). Five specimens of each material were weighed every fourth cycle.

#### 2.2.3. Thermogravimetric Analysis

Thermal stability was evaluated using thermogravimetric analysis (TGA; Mettler Toledo TGA/DSC3+, Mettler Toledo GmbH, Switzerland) under a nitrogen environment, with heating from 25 °C to 800 °C at a rate of 10 °C/min. Three specimens per material (8–12 mg) were tested after every fourth cycle.

#### 2.2.4. Mechanical Properties

Short-term creep was assessed at 25 °C using a DMA Q800 (TA Instruments, New Castle, DE, USA) in a single cantilever configuration. A 4 MPa load was applied for 3 min, followed by a 10 min recovery period (60 min for the final cycle). Three specimens of each material were tested in the non-aged state and after twelve aging cycles.

The flexural modulus was measured by three-point bending (Instron 4411, Instron Corp., Norwood, MA, USA; ASTM D790-25 [41]) on specimens (80 × 12 × 4 mm^3^) at 1.71 mm/min. Five specimens per material were tested every fourth cycle.

Fracture toughness (K*_1c_*) was determined in single-edge notch-bend tests (Instron 4411, ASTM D5045-14 [42]) at 25 °C on specimens (52.5 × 12 × 6 mm^3^, five specimens per material) that were notched with a razor blade and loaded at 10 mm/min. Tests were conducted every sixth cycle.

#### 2.2.5. Tribological Characterisation

Tribological behaviour was characterised using a ball-on-disk configuration with a 10 mm stainless-steel 316L ball, in air (after every complete fourth cycle of cryogenic cyclic aging) and in vacuum (after twelve cycles), at 25 °C. The initial roughness (*R_a_*) of the countersurface and a polymer disk was 0.1 μm and 0.2 μm, respectively. Other test parameters are summarised in Table 2.

A single load and sliding speed were used to isolate the effects of cryogenic cyclic aging and to allow a direct comparison with the non-aged baseline materials reported previously [43].

#### 2.2.6. Fracture and Wear Surface Characterisation

Wear tracks were analysed by white-light interferometry (Zygo NewView 7200, Zygo Corporation, Middlefield, CT, USA), using the average of four cross-sections to calculate the wear rate (mean of three tests).

Fracture and wear surfaces were imaged by scanning electron microscopy (SEM) (JEOL JSM-IT30, JEOL Ltd., Tokyo, Japan) after platinum coating to improve their conductivity during the SEM analysis.

For a more detailed description of the methodology, the reader is referred to our previous publication [43].

#### 2.2.7. Statistical Analysis

Statistical analysis was used to assess the significance of cryogenic cyclic aging regarding the measured properties. Welch analysis of variance (ANOVA) was applied, where several aging conditions were compared. Post hoc comparisons (a pairwise Welch *t*-test with Holm correction) were used to identify significant differences between individual aging conditions. Where only two conditions were compared, Student’s *t*-test was used. The significance level was set at *p* < 0.05. Changes with *p* ≥ 0.05 were treated as statistically non-significant and are discussed as trends only.

## 3. Results and Discussion

### 3.1. Weight Characterisation

**Figure 2:**
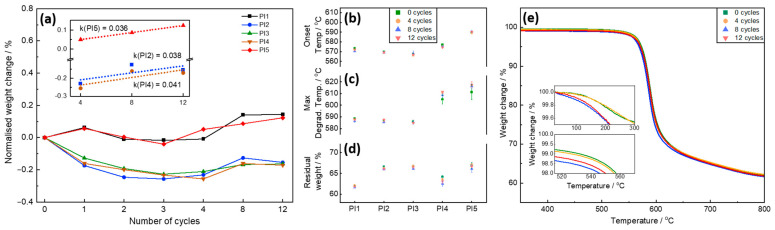
Effects of cryogenic cyclic aging on specimens’ (**a**) weight, (**b**) degradation onset temperature, (**c**) peak degradation temperature, and (**d**) residual weight and (**e**) TGA curves of PI1. Values at 0 cycles correspond to non-aged materials.

Figure 2a shows the variation in specimen weight, normalised to the initial weight of the non-aged material, with increasing aging time. Although the weight varied by no more than 0.25%, the ANOVA (Table 3) confirmed that the effect of cryogenic cyclic aging on the normalised weight was statistically significant for the investigated materials. The analysis supported a slight but significant increase in weight for the unreinforced polyimides, PI1 and PI5. In contrast, the PI composites showed an initial reduction in normalised weight after the first aging cycles, followed by a partial increase toward the 12th cycle. The decrease in weight was likely associated with the detachment of some polymer or filler lumps caused by rapid physical shrinkage and variations in the coefficients of thermal expansion between the matrix and filler when the composites were immersed in liquid nitrogen. Debris were found at the bottom of the container after the cyclic aging of the specimens. It is noteworthy that the linear fit of the experimental data revealed similar slope values (*k*, Figure 2a) for the unreinforced polyimide and composites. The increasing weight may be caused by polymer shrinkage at cryogenic temperatures and structural rearrangements, partially remaining after returning to 25 °C, which results in enhanced moisture adsorption, as shown in Figure 2e and discussed below [44,45].

### 3.2. Thermogravimetric Analysis

**Figure A1:**
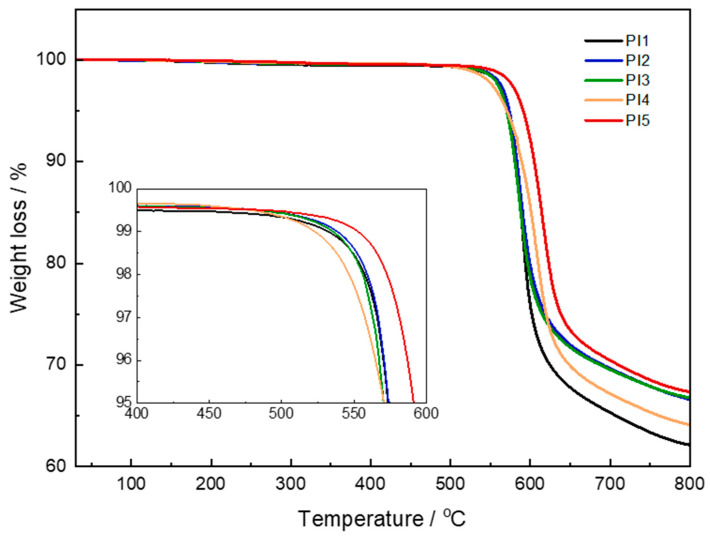
TGA curves of non-aged PI-based materials.

The thermogravimetric analysis revealed that the thermal degradation behaviour of the PI materials depended on the composition (Figure 2b–d). The onset temperature (Figure 2b) marks the start of degradation, and differences in onset were attributed to differences in polyimide structure. Superior thermal stability was demonstrated by PI5, increasing the degradation temperature by 15 °C compared to other polyimides. Polyimides with more rigid chains tend to decompose at higher temperatures, which is assumed to be the case for PI5 [46,47,48]. Adding fillers changed the degradation temperatures only slightly, by 2–4 °C (Figure 2b), but the residual weight at 800 °C was higher than that of PI1 (Figure 2d). When polyimide was reinforced with graphite and PTFE particles, degradation was initiated at lower temperatures (Appendix A, Figure A1) because PTFE is less thermally stable than polyimide [49]. At the same time, the onset temperature of PI4 was increased by 3 °C compared to PI1, and the decomposition rate of PI4 was lower compared to PI3, resulting in a higher temperature of maximum degradation (Figure 2c). The products of PTFE decomposition might act as barriers, decreasing the decomposition rate of PI4 [50].

Cryogenic cyclic aging had only a minor effect on the thermal stability of the tested polyimide-based materials (Figure 2b–e). No variations in onset temperature, maximum degradation temperature, or residual weight were detected for the PI1, PI2, and PI3 materials. The degradation rate was slower for PI4 and PI5 after cryogenic cyclic aging, resulting in an insignificantly increasing temperature of maximum degradation. At the same time, cryogenic cyclic aging was responsible for a slight reduction in residual weight for PI4 and PI5. This might be due to the incomplete relaxation and residual physical shrinkage of polyimide chains, as in the case of PI5, and the effects of cryogenic cyclic aging mainly on PTFE particles, as in the case of PI4, since such changes were not observed for polyimide reinforced with graphite only (PI3). 

It is worth noting that the weight loss of the cryogenic cyclic-aged specimens began to deviate from that of the non-aged specimen above 40 °C, and the deviation became more evident with an increasing number of aging cycles (Figure 2e). A higher deviation rate was observed after 100 °C, with the difference in weight loss between 12-cycle-aged and non-aged specimens reaching almost 0.8% at the onset temperature. The variation in weight loss in the range of 40 °C and 100 °C was attributed to the evaporation of moisture absorbed by the surface. The continuing increase in weight loss deviation may be evidence of contamination, which varied between the specimen batches.

### 3.3. Mechanical Properties

#### 3.3.1. Short-Term Creep

**Figure 3:**
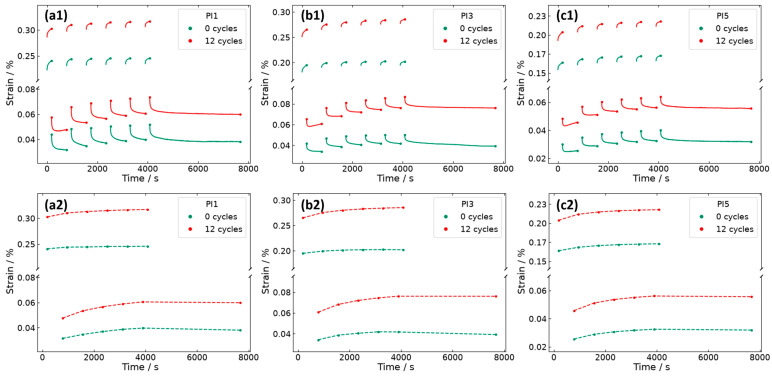
Short-term creep results of (**a**) PI1, (**b**) PI3, and (**c**) PI5, measured before aging (0 cycles) and after 12 cycles at 25 °C under 4 MPa load. Figures with label (**1**) indicate the strain response of the materials, and those labelled (**2**) show trends in creep and residual strain developed over time. Note that scale values differ between the materials.

**Figure A2:**
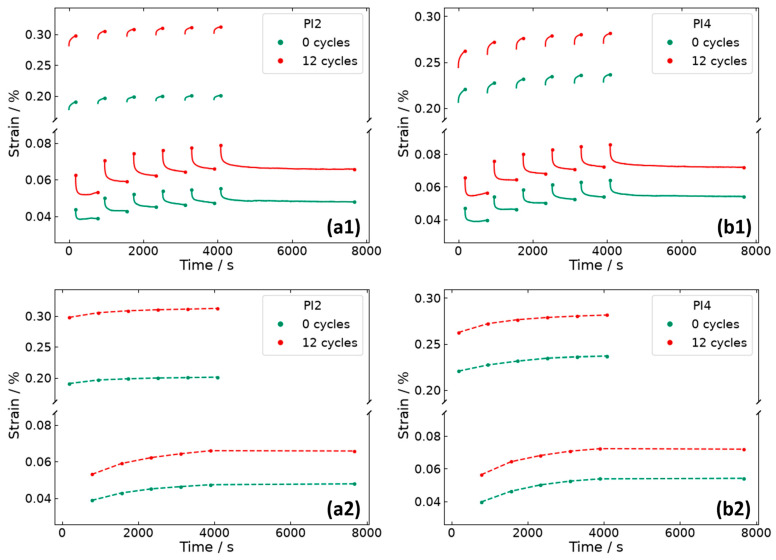
Experimental short-term creep results of (**a**) PI2 and (**b**) PI4, measured before aging and after 12 cycles at 25 °C under 4 MPa load. Figures with label (**1**) indicate the strain responses of the materials, and those labelled (**2**) show trends in creep and residual strain developed over time. Note that scale values are varied for the materials.

The effect of cryogenic cyclic aging on the viscoelastic behaviour of the polyimide specimens was evaluated by a short-term creep test. Figure 3 presents the creep and relaxation curves for PI1, PI3, and PI5. PI2 and PI4 exhibit similar trends and are shown in Appendix A, Figure A2. The creep and residual strain in both non-aged and aged specimens increased with the number of short creep cycles [51]. The greatest creep and residual strains occurred during the first loading step, after which the rate of strain accumulation progressively decreased in subsequent steps [43].

The superior mechanical properties of PI5 and the composites resulted in their improved creep resistance compared to PI1. In particular, the elastic strain or strain instantly induced in the materials after loading was lower than that of PI1. Similarly, the creep strain at the end of short-term creep was reduced by approximately 18% for PI2 and PI3 and 30% for PI5. Creep and residual strain develop through the extension and reorientation of polymer chains under a constant load [51], so that the accumulated stress can lead to irreversible deformation or chain rupture while the polymer is loaded. Adding stiff fillers or using more rigid chains limits chain movement and improves creep resistance. Reinforcing the polyimide with PTFE particles, which have poorer mechanical properties than the matrix [15], increased the creep and residual strain (Appendix A, Figure A2). Even though the addition of fillers enhanced the stiffness of the composites and constrained polymer chain movement, one may notice that the residual strain remaining after the relaxation phase was slightly higher relative to PI1, likely due to stress accumulation, mainly at the undefined filler/matrix interface.

Cryogenic cyclic aging clearly affected the creep behaviour of the polyimide, reducing its creep resistance. At the last creep step, the creep strain increased by 29% for PI1, 57% for PI2, 40% for PI3, 18% for PI4, and 20% for PI5 relative to the non-aged specimens, while the residual strain at the end of the test increased by 57%, 38%, 94%, 32%, and 74%, respectively. Interestingly, the 12 cycle-aged specimens gave equal creep and residual strains, i.e., for PI1 and PI2 (about 0.31% and 0.06%) and for PI3 and PI4 (0.28% and 0.07%). The increased strain is probably linked both to structural changes, mainly in the unreinforced polyimides PI1 and PI5, and to the difference in thermal expansion between the matrix and filler, which drives the accumulation of thermal stress at the filler/matrix interface. From these results, the shrinkage of the polyimide appears to dominate in PI1 and PI2, whereas the stress state at the graphite/polyimide interface controls strain development in PI3 and PI4.

#### 3.3.2. Effects of Cyclic Aging on Polymer Structure

The thermal and short-term creep results suggest that cryogenic cyclic aging causes physical changes in the polyimide structure through the shrinkage of the polyimide molecules at a low temperature. Some polyimides also undergo low-temperature relaxations that can lead to structural rearrangement [52,53]. Chopra et al. [45] showed that amorphous regions in ultra-high-molecular-weight polyethylene can undergo cryo-structural rearrangement, forming a more oriented chain structure and changing the crystallinity. In addition, repeated cycles from −196 °C to +40 °C can build up thermal stress and deform the chains; if this stress exceeds a critical value, chain rupture and permanent plastic deformation can occur. The chosen relaxation time (one day) and temperature (40 °C) may have been too short or too low to fully relieve the residual stress in the polyimide. As a result, fewer molecular chains were able to carry the applied stress, which rendered chain extension, material deformation, and microcracking easier (polyimide has low fracture toughness) [37].

**Figure 4:**
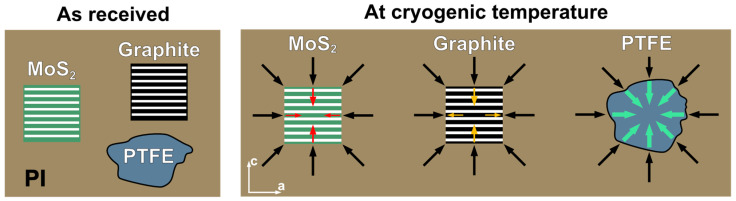
Schematic visualisation of polyimide and filler shrinkage at cryogenic temperature. Arrow thickness and orientation illustrate the varying magnitude and direction of CTE changes. Arrow colours correspond to the different materials: black for PI, red for MoS_2_, yellow for graphite, and green for PTFE.

As previously mentioned, fillers or more rigid chains can limit chain/molecular mobility and reduce physical shrinkage. However, stress accumulation at the filler/matrix interface, affected by the CTE differences between the polyimide and filler, may locally alter the stress state and cause crack generation and the propagation of microcracks. Figure 4 schematically presents the direction and magnitude of changes in CTE between the constituents. At cryogenic temperatures, graphite is known to contract in the c-direction (perpendicular to the basal planes) and has a negative CTE in the a-direction (basal plane) [54], while MoS_2_ shrinks in both directions [55]. However, the CTE values of graphite and MoS_2_ are lower than those of polyimides [56], leading to the accumulation of residual compression stresses at the filler/matrix interface. In contrast, PTFE exhibits a higher CTE than polyimide, which may cause debonding at the interface [57].

#### 3.3.3. Flexural Modulus

**Figure 5:**
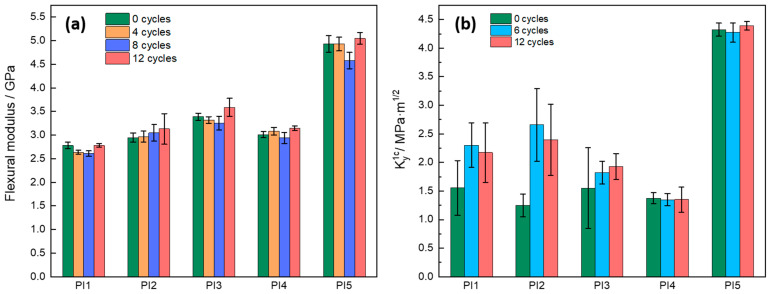
(**a**) Flexural modulus and (**b**) fracture toughness of PI-based materials following cryogenic cyclic aging, both evaluated at 25 °C. Values at 0 cycles correspond to non-aged materials.

The flexural moduli of non-aged and cyclic-aged materials are presented in Figure 5a. The variation in the flexural modulus for all tested materials was within 8%, indicating a minor effect of cryogenic aging on flexural properties.

The contrasting responses regarding the flexural modulus and the creep compliance can be explained by the different deformation regimes that they reflect. The flexural modulus reflects the instantaneous, primarily elastic response governed by the covalent backbone and short-time chain packing and is therefore largely unaffected by aging. Creep compliance is a time-dependent viscoelastic property governed by segmental mobility, free volume, and secondary relaxations. As discussed above, cryogenic cyclic aging leaves the polyimide chains in an incompletely relaxed, non-equilibrium state with residual stresses and locally increased free volume. Under a sustained load, this promotes segmental rearrangement and chain extension, raising the time-dependent strain (Figure 3) while leaving the instantaneous bulk modulus essentially unchanged.

Similarly to polyimide-based composites, other researchers [58,59] have reported that the flexural and tensile properties of epoxy-based materials are not significantly affected by short-term cryogenic aging. In contrast, Dasari et al. [29,30] and Whitley et al. [31,32] revealed improved flexural and tensile properties for epoxy- and polyimide-based laminates reinforced with carbon fibres after short- (up to 16 h) and long-term (596 h) cryogenic aging, respectively. The improved mechanical properties at longer aging times were attributed to the recovery of the thermal stresses generated in the polymer due to thermal shock.

In our study, microstructural changes did not evolve into a decrease in mechanical properties at the macroscale. It is likely that, if the cyclic aging was continued for a longer period of time, microstructure changes would be discovered at the macroscale as well, as was noticed by Kumar et al. [60].

#### 3.3.4. Fracture Toughness

The influence of cryogenic cyclic aging on the fracture toughness values (*K*_1*c*_) of the polyimides is presented in Figure 5b, and the corresponding ANOVA results are summarised in Table 4. Although the *K*_1*c*_ values had increased already after six cycles of cryogenic aging for PI1, PI2, and PI3, the relatively high standard deviations indicate considerable scatter, most likely associated with specimen inhomogeneity and the sensitivity of fracture toughness measurements to notch preparation and the local microstructure. After 12 cycles, the average increase in *K*_1*c*_ reached 40% for PI1 and 93% for PI2 compared with the non-aged specimens, while PI3 showed a smaller increase of up to 24%. The fracture toughness was not altered for the PI4 and PI5 materials. The ANOVA confirmed the statistically significant effect of cryogenic cyclic aging only for PI2. For PI1, PI3, PI4, and PI5, the changes were not statistically significant and should therefore be interpreted as trends rather than confirmed effects. Contrarily, in the authors’ previous work [37], the fracture toughness of neat polyimides was found to decrease after long-term cryogenic aging.

**Figure 6:**
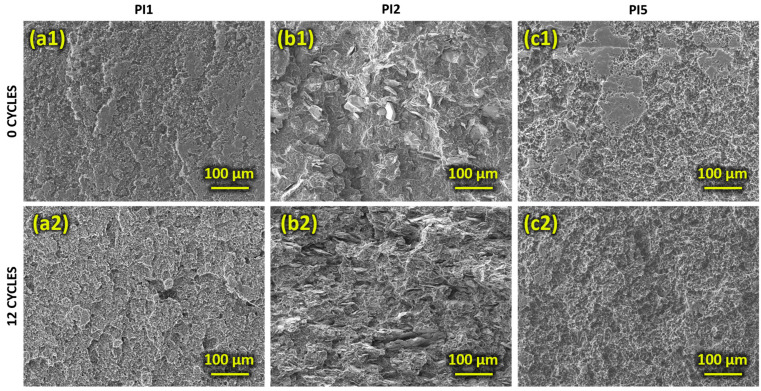
Fracture surface morphologies of PI materials tested at 25 °C: (**1**) non-aged (0 cycles) and (**2**) after 12 cryogenic aging cycles—(**a**) PI1, (**b**) PI2, and (**c**) PI5. Fracture images of non-aged (0 cycles) specimens are adapted from [37].

**Figure 7:**
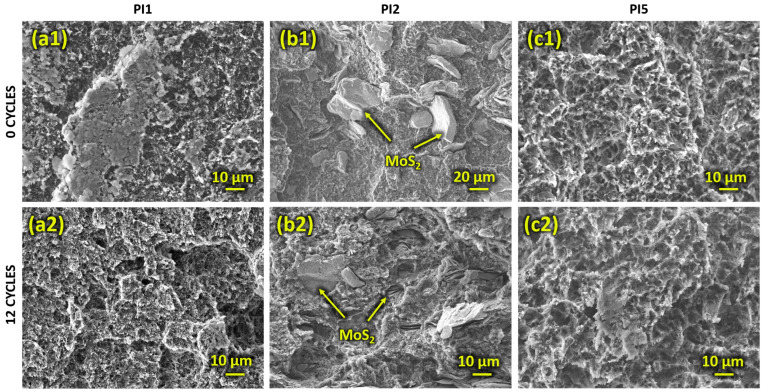
High-magnification fracture surfaces of PI materials tested at 25 °C, comparing (**1**) non-aged (0 cycles) and (**2**) those after 12 cryogenic aging cycles: (**a**) PI1, (**b**) PI2, and (**c**) PI5. Fracture images of non-aged (0 cycles) specimens are adapted from [37].

**Figure A3:**
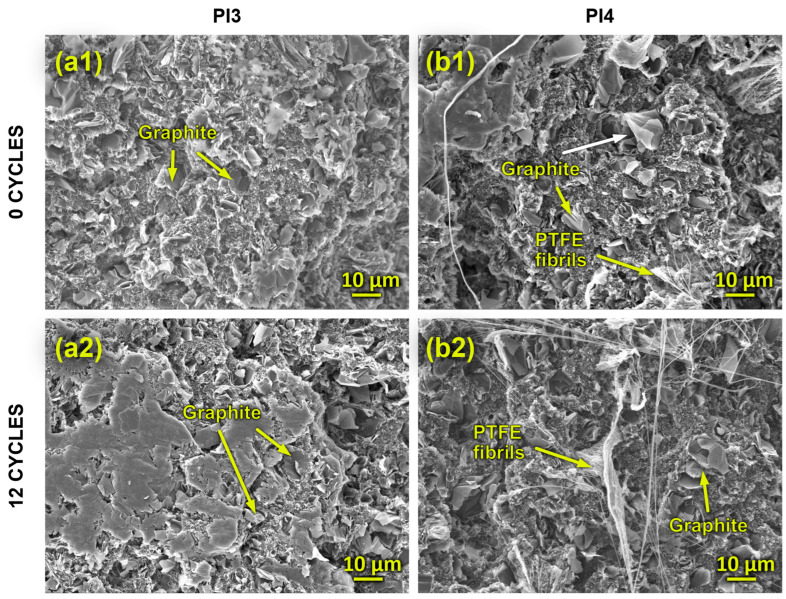
Fracture surfaces of (**1**) non-aged and (**2**) 12-cycle-aged (**a**) PI3 and (**b**) PI4 tested at 25 °C. Figures of non-aged materials adapted from [37].

Figure 6 and Figure 7 present the fracture surfaces of the non-aged and cyclic-aged polyimides tested at 25 °C (fracture surfaces of PI3 and PI4 are shown in Appendix A, Figure A3). A quasi-ductile fracture mode persisted after cryogenic cyclic aging, exhibiting a notably rougher fracture surface compared to the non-aged specimens. As discussed previously, the repeated aging cycles introduced thermal stresses in the polyimide, which possibly reduced the mobility of molecular chains and/or initiated structural rearrangements in the polyimide. The incomplete relaxation of the polyimide resulted in an increase in energy required to initiate crack propagation.

**Figure 8:**
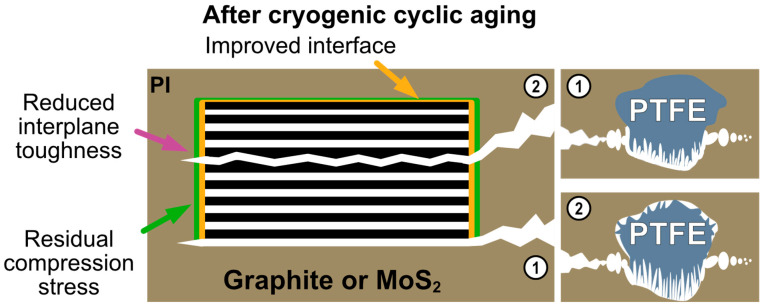
Schematic of changes at the filler/matrix interface after cryogenic cyclic aging. Direction (1) represents the non-aged materials, and direction (2) indicates the changes after cyclic aging.

The fracture surfaces of the composites (Figure 6b and Figure 7b, Figure A3 in Appendix A) do not demonstrate any notable differences compared to the non-aged materials. In this case, the fracture toughness is assumed to be governed by the previously explained toughening mechanism of the matrix. Variations in the *K*_1*c*_ values among the composites are likely attributed to differences in stress concentration at the filler/matrix interface (Figure 4) due to the thermal cycling, in line with observations by other researchers [29,33,60]. Figure 7(b2) shows more frequent separation in MoS_2_ in the cryogenic cyclic-aged specimen. During aging in liquid nitrogen, the interface is likely improved, along with the introduction of thermal stresses due to the different CTEs between the filler and the matrix (Figure 8) [55,61]. In non-aged composites, a crack likely propagates in the direction indicated by number 1 in Figure 8, through an undefined filler/matrix interface. Repeated thermal cycles may reduce the interplanar strength of the filler (Figure 7(b2) and Figure A3(a2,b2)). Interestingly, transparticle debonding or improved graphite/polyimide interfaces were not observed in the cyclic-aged PI3 and PI4. In the case of PTFE (Appendix A, Figure A3b), the initially cohesive PTFE/polyimide interface and the repeated shrinkage/expansion of the filler may have led to the increased tearing of PTFE particles, forming voids or a low-strength interface that acted as stress concentrators (Figure 8, direction 2; Figure A3(b2)). After cryogenic cyclic aging, cracks therefore tend to develop both in direction 1 (favoured by the high residual stress, especially at the graphite/polyimide interface) and in direction 2 (Figure 8), where the interplanar or interfacial strength is low. On the one hand, this debonding and the thermal stress should lower the fracture toughness and the interface strength, reducing stress transfer and increasing the residual stress in the matrix (Figure 3). On the other hand, the toughening of the matrix and the dissipation of crack energy through multiple interplanar debonding lead to ductile fracture and slower crack propagation, which raises the fracture toughness.

### 3.4. Tribological Characterisation

#### 3.4.1. Friction and Wear in Air at 25 °C

**Figure 9:**
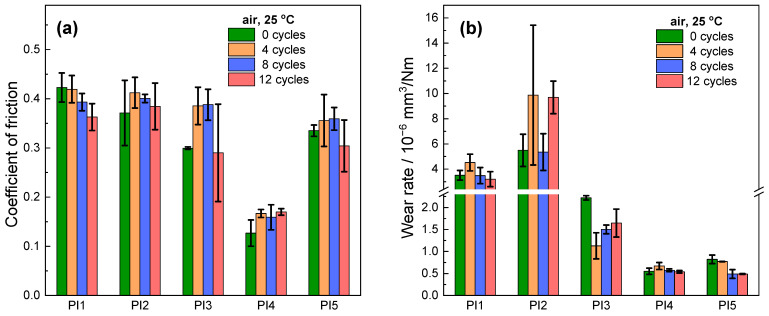
(**a**) Coefficients of friction and (**b**) wear rates of PI materials before and after cryogenic cyclic aging when tested in air at 25 °C. Values of non-aged (0 cycles) materials are from [37].

The coefficients of friction and wear rates of the non-aged and cryogenic cyclic-aged specimens are presented in Figure 9, while the corresponding ANOVA results are summarised in Table 5. The polyimide structure and the reinforcements both significantly contributed to the changes in CoF (Figure 9a) and wear rate (Figure 9b) after cryogenic cyclic aging. PI1 demonstrated a trend of decreasing CoF with an increasing number of cryogenic cycles, reaching a 14% reduction after 12 cycles. PI3 exhibited a statistically significant change in CoF during cyclic aging, with the average CoF increasing after four cycles and subsequently decreasing by approximately 25% between 8 and 12 cycles. However, the post hoc paired *t*-tests with Holm correction did not identify any individual pairwise differences that remained statistically significant, likely due to the small sample size available to identify specific pairwise differences after correction for multiple comparisons (Appendix A, Table A1). PI4 also showed an average CoF increase after four cycles, reaching approximately 31%, but this variation was not statistically significant. A minor alteration in the coefficient of friction with an increasing number of cryogenic aging cycles was found for PI2 and PI5.

The wear rate of the materials (Figure 9b) was less affected by cryogenic cyclic aging than the coefficient of friction. One may notice that a transition in tribological behaviour occurred after four aging cycles. PI1 and PI4 exhibited the smallest variations in wear rate, despite a 28% increase in wear rate for the PI1 material after four aging cycles. The PI2 material experienced large fluctuations in wear rate, which subsequently showed a rise of 77%, compared to the non-aged materials. However, the ANOVA revealed a *p*-value of 0.06, indicating no significant differences in the wear rate. The high standard deviation was assumed to occur due to inhomogeneity among the batches of specimens, as was mentioned previously. Cryogenic cyclic aging reduced the wear rates of PI3 and PI5, with the decrease reaching 26% and 41% after 12 cycles. Although there are limited studies that have investigated the effects of cryogenic aging on the tribological performance of PI-based materials, authors [62,63] generally report improved hardness and reduced abrasive wear for various polymers after cryogenic aging. Similarly to the observations in the present study, Indumathi et al. [62] revealed the improved performance of cryogenic cyclic-aged PI reinforced with PTFE (PI3) or PTFE and graphite (PI4), while the addition of MoS_2_ (PI2) did not substantially affect the wear rate of the composite.

**Figure 10:**
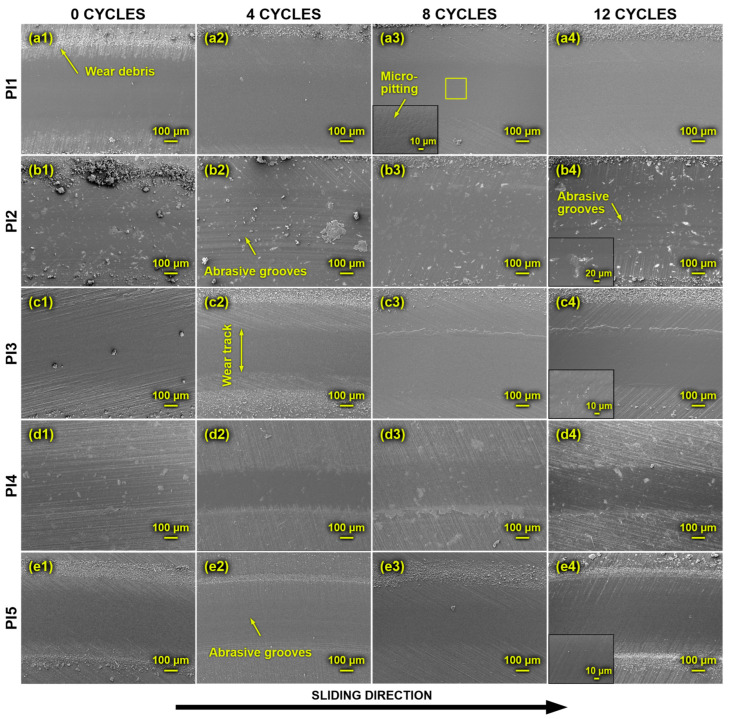
Wear tracks of (**a**) PI1, (**b**) PI2, (**c**) PI3, (**d**) PI4, and (**e**) PI5 after (**1**) 0 cycles, (**2**) 4 cycles, (**3**) 8 cycles, and (**4**) 12 cycles of cryogenic aging when tested in air at 25 °C. Figures of non-aged (0 cycles) materials adapted from [37]. The yellow box indicates the region magnified in the inset.

The evolution of the wear tracks of the studied materials with an increasing number of cryogenic aging cycles is shown in Figure 10. The wear tracks of the cyclic-aged unreinforced polyimides, PI1 and PI5, did not exhibit any notable changes compared to the non-aged materials. However, abrasive wear became more pronounced with the increasing duration of the cyclic aging experiment (Figure 10e). Fine wear debris and ball asperities are likely responsible for the generation of abrasive micro-grooves on the disk. Due to the improved fracture toughness after cryogenic cyclic aging, the micro-pitting found on the non-aged PI1 [37], and remaining after eight aging cycles (Figure 10(a3)), was mitigated after 12 cycles, resulting in a smooth wear track.

**Figure 11:**
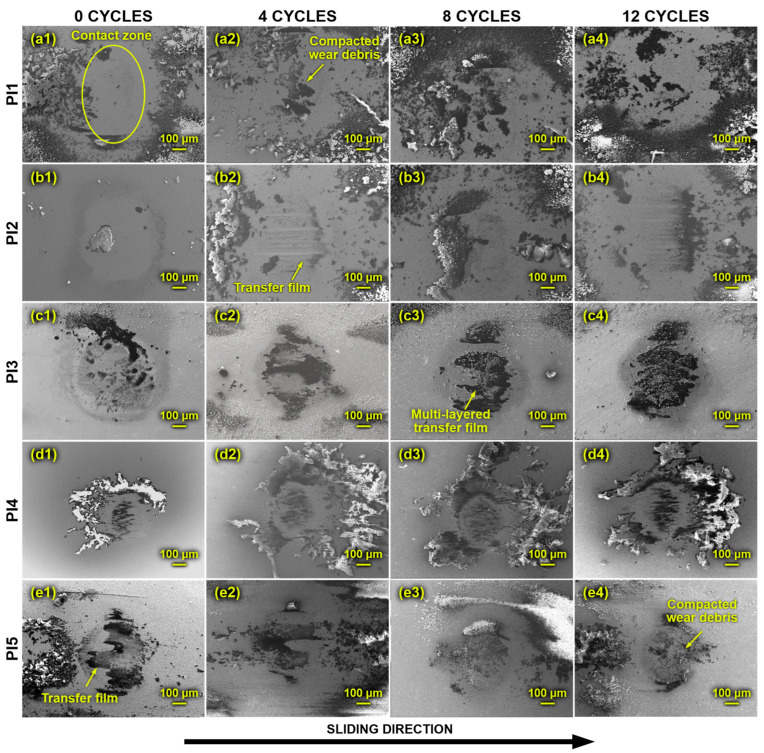
Countersurfaces of (**a**) PI1, (**b**) PI2, (**c**) PI3, (**d**) PI4, and (**e**) PI5 after (**1**) 0 cycles, (**2**) 4 cycles, (**3**) 8 cycles, and (**4**) 12 cycles of cryogenic aging when tested in air at 25 °C. Figures of non-aged (0 cycles) materials adapted from [37].

A triboreaction between the polar carbonyl groups of the polyimide and the metal surface, forming a metal–radical bond, is known to produce a thin, oriented transfer film on the metal countersurface [2,64,65]. One may notice that cryogenic cyclic aging hindered the formation of a thin transfer film (compare Figure 11(a1) and Figure 11(a4), as well as Figure 11(e1,e4)). Despite the higher fracture toughness, the greater residual stress (Figure 3) and the reduced chain mobility after aging appear to have changed how energy is dissipated and how the transfer film forms in the contact. Rolled and flake-like wear debris formed earlier than the frictional softening and plastic deformation needed to produce a thin transfer film, as seen in the non-aged specimens (Figure 11(e1)) [37]. Although some shearing of wear debris was still found in the contact zone, compacted wear debris formed polymer patches on PI1 (Figure 10(a4)) and on the countersurfaces sliding against PI1 (Figure 11a) and PI5 (Figure 11e). This layer of compacted wear debris played the role of a barrier, protecting the virgin polymer surface from excessive wear. A similar effect was observed with the addition of fillers to the polyimide matrix [66,67]. The reduction in the CoF was due to the decreased impact of adhesive friction compared to the non-aged specimens.

The wear tracks of the aged composites (Figure 10b–d) do not demonstrate significant differences compared to the non-aged specimens [37]. However, a few differences still exist. The fracture toughness was found to control the wear rate of the composites. The presence of more ploughing and abrasive grooves on the wear track of PI2 after four (Figure 10(b2)) and eight cycles (Figure 10(b3)) of cryogenic aging correlates with the increase in wear rate (Figure 9b). When the fracture toughness was not sufficient, the formation of fine and flake-like wear debris originating from the chipping and stretching of the polyimide was observed on the wear track (Figure 10(b1,b2)). With increasing fracture toughness after cryogenic cyclic aging, debonding between the fillers and matrix does not occur until the residual stresses or stresses at the filler/matrix interface reach critical values [68]. Exceeding the critical stresses leads to the release of single MoS_2_ platelets due to reduced particle toughness (Figure 7(b2)). The plastic deformation of the polyimide and the easy release of MoS_2_ particles resulted in the formation of an inhomogeneous transfer film and abrasive molybdenum oxide particles, increasing the abrasive wear of the composite and countersurface (Figure 11(b2–b4)) [17,69]. In some parts of the wear track, wear debris was collected in the holes, generated by the partial or total removal of MoS_2_ particles (Figure 10(b4)).

More fine and plate-like wear debris were observed at the wear track edges of PI3 and PI4 (Figure 10c,d), respectively, with an increasing duration of cryogenic cyclic aging. However, notable changes were found on the countersurfaces sliding against these materials. With an increasing number of cryogenic aging cycles, the formation of the transfer film was enhanced. In the case of the PI3 material, the inhomogeneous and patchy transfer film of the non-aged specimen (Figure 11(c1)) was transformed into a multilayered transfer film after 12 cycles of aging (Figure 11(c4)). The first layer comprised polymer lumps, while the second layer consisted of compressed flake-like wear debris. The morphology of the PI4 transfer film (Figure 11d) was not significantly changed, except for an increased area covered by the transfer film and wear debris accumulated around it. As discussed previously, cryogenic cyclic aging likely led to the more intensive fibrillation of PTFE particles and the greater accumulation of residual stresses at the graphite/polyimide interface. In turn, this facilitated the easy release of the fillers from the matrix, thereby increasing the amounts of graphite and PTFE in the surface layers. The lubricity of graphite and PTFE in this case is not mitigated by the matrix trapping effect [18]. The formed transfer films likely had a higher thickness, sufficient to reduce the wear rate of the cyclic-aged PI3 and PI4, acting as a barrier between the abrasive wear debris and ball asperities. However, the CoFs of these materials were increased due to elevated adhesive forces. Interestingly, a reduction in wear rate and a simultaneous increase in the CoF of PI3 (Figure 9) were observed after four cycles of cryogenic aging. The subsequent accumulation of a multilayered transfer film (Figure 11(c3,c4)) with an increasing duration of cryogenic aging resulted in a reduction in the CoF due to the decreased shear strength of the film. Oppositely, this transfer film likely increased the adhesive and fatigue wear of the composite, increasing the wear rate after four cycles of cryogenic cyclic aging. The variations in the CoF and wear rate were minimal in the case of the addition of PTFE due its band structure, which allowed the formation of transfer films with low shear strength [70,71]. Additionally, to the authors’ knowledge, there are no published papers that have investigated the behaviour of polyimide composites after cryogenic cyclic aging. However, similarly to the observations of non-aged materials in [13,72,73], graphite’s addition to the polyimide was not advantageous in producing a transfer film capable of CoF reduction.

#### 3.4.2. Friction and Wear in Vacuum at 25 °C

**Figure 12:**
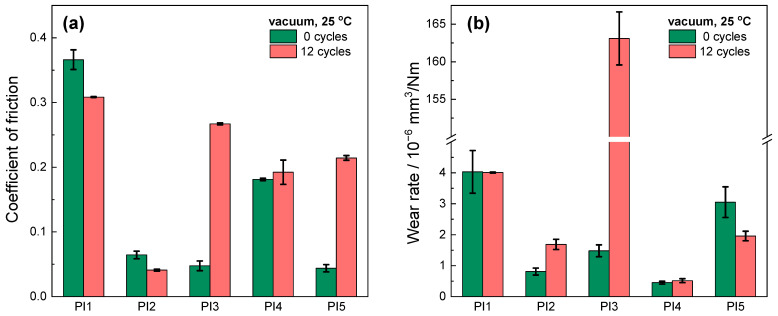
(**a**) Coefficients of friction and (**b**) wear rates of PI materials before and after cryogenic cyclic aging when tested in vacuum at 25 °C. Values of non-aged (0 cycles) materials are from [37].

The effects of cryogenic cyclic aging on the tribological performance in vacuum differed from that in air. The changes in the coefficient of friction and wear are presented in Figure 12.

**Figure A4:**
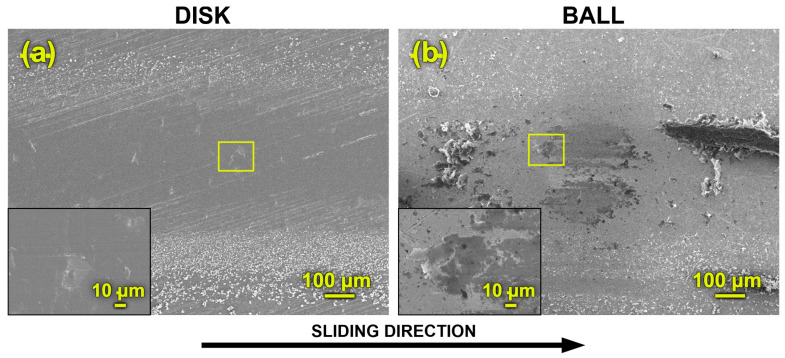
(**a**) Wear track and (**b**) countersurface of PI4 after 12 cycles of cryogenic aging when tested in vacuum at 25 °C. Figures of non-aged materials adapted from [37]. The yellow box marks the region shown at higher magnification in the inset.

In a vacuum, the CoF decreased by 17% for PI1 and 30% for PI2 after 12 cycles. PI3 and PI5, by contrast, showed large increases in the CoF of 550% and 425%, respectively, relative to the non-aged materials. The wear rate of PI1 remained unchanged after cryogenic aging, while the wear rate of PI5 was reduced by 35%. An increase in wear rate of 107% and a significant increase of two orders of magnitude (10,920%) was found for PI2 and PI3, respectively. Similarly to the observations in air, the smallest variations in the CoF and the wear rate were shown by PI4 due to the formation of a transfer film with low shear strength, which prevented significant changes in wear mechanism [37]. SEM images of PI4’s wear track and countersurface are presented in Appendix A, Figure A4.

**Figure 13:**
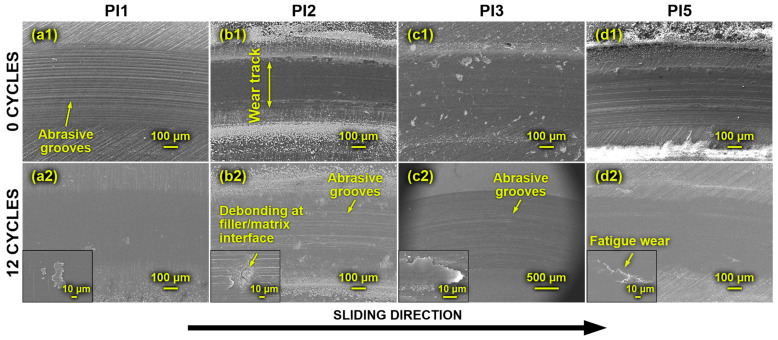
Wear tracks of (**a**) PI1, (**b**) PI2, (**c**) PI3, and (**d**) PI5 after (**1**) 0 cycles (non-aged) and (**2**) 12 cycles of cryogenic aging when tested in vacuum at 25 °C. Figures of non-aged (0 cycles) materials adapted from [37].

**Figure 14:**
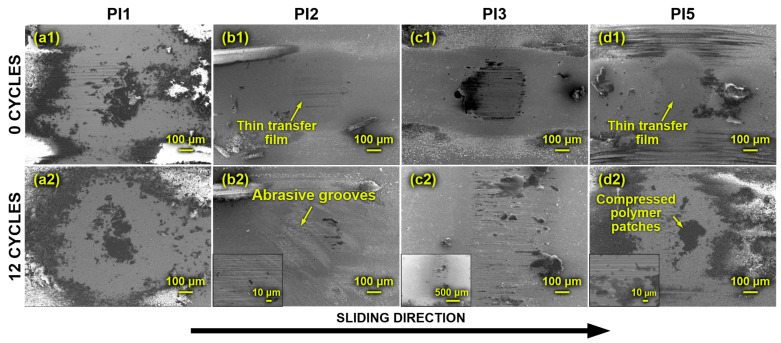
Countersurfaces of (**a**) PI1, (**b**) PI2, (**c**) PI3, and (**d**) PI5 after (**1**) 0 cycles (non-aged) and (**2**) 12 cycles of cryogenic aging when tested in vacuum at 25 °C. Figures of non-aged (0 cycles) materials adapted from [37].

The wear tracks of the cryogenic cyclic-aged unreinforced polyimides, PI1 (Figure 13a) and PI5 (Figure 13d), became smoother, with the absence of notable ploughing grooves found for non-aged materials. Wear debris compacted on the countersurfaces of PI1 (Figure 14(a2)) and PI5 (Figure 14(d2)), forming a lumpy transfer in the contact zone.

As discussed previously, if the mobility of polyimide molecular chains is sufficient, polyimides form a thin transfer film on the countersurface oriented in the sliding direction [1,2]. In vacuum, frictional heat can be dissipated only through the thermal conductivity of the materials, and the absence of water molecules lowers the shear strength of the polyimide and increases plastic deformation. However, the incomplete relaxation of the molecular chains reduced the ability of the material to form a thin oriented transfer film on the countersurface, as was observed for the non-aged PI5 (Figure 14(d1)). Additionally, the accumulation of residual stresses (Figure 3) changed the energy dissipation mechanism, producing fine and flake-like wear debris, before the polyimide underwent sufficient plastic deformation. Due to the increased fracture toughness, the abrasiveness of small wear debris was reduced, preventing grooving and resulting in the polishing of PI1 and PI5’s wear tracks. As was mentioned earlier, some researchers [45,62,63] have revealed the increased hardness of polymers after cryogenic treatment, which resulted in their improved resistance to abrasive wear.

At the same time, the wear tracks and countersurfaces of the composites indicated the opposite mechanism. The wear track of PI2 demonstrated increased abrasive wear with the formation of rolling pits on the polymer disk (Figure 13(b2)). Due to incomplete relaxation and the accumulation of residual stresses after cryogenic cyclic aging, the delamination between MoS_2_ particles and the polyimide was more pronounced. Similarly to the observations in air, the abrasive marks were present more frequently on the countersurface (Figure 14(b2)), likely originating from the formation of small wear debris at the interface and the release of single MoS_2_ platelets scratching the surface. The reduction in adhesive forces and in plastic deformation was likely responsible for the decrease in CoF.

Cryogenic cyclic aging had a detrimental effect on the tribological performance of the graphite-filled polyimide (PI3) in a vacuum. The width of the wear track (Figure 13(c2)) of the cryogenically aged PI3 was 2.5 times wider than that of the non-aged materials. Fatigue and abrasive wear were significantly increased, generating plate-like wear debris due to the spalling of the thick surface layers. Small wear debris, originating from the crushing of graphite particles, and strain-hardened polyimide wear debris abraded the polymer disk and the countersurface (Figure 14(c2)). The contact zone on the countersurface had an oval shape, compared to the smaller circular shape in the case of the non-aged PI3. Peeled polymer lumps adhered to the countersurface, forming a loosely attached transfer. The deterioration of the tribological performance of the cryogenic cyclic-aged PI3 could be attributed to the influence of several factors. First, as was discussed previously, the incomplete relaxation of the molecular chains and the accumulation of residual stresses (Figure 3) reduced the ability of the material to plastically deform in the vacuum environment. The SEM observations suggest that, instead of forming a thin and stable transfer film through shear-induced plastic deformation, the aged surface became more susceptible to stress concentration, crack initiation, and delamination. When the local stresses exceeded a critical level, microcracks propagated within the near-surface region, leading to the delamination of the polymer surface and the formation of plate-like wear debris. Second, the fracture surface observations suggest that cryogenic cyclic aging may have modified the graphite/polyimide interface (Appendix A, Figure A3a). Although improved interfacial contact may promote stress transfer during fracture, the graphite/polyimide interface may still facilitate particle release through transparticle debonding. Once trapped in the contact, graphite particles may contribute to three-body abrasion rather than forming a continuous lubricating transfer layer, especially together with strain-hardened polyimide debris. Another possible factor is that graphite’s lubricity may be reduced in a vacuum because dangling bonds are not fully saturated. Even though polyimides can absorb water molecules from the environment [2] and may saturate graphite dangling bonds with desorbed gases in a vacuum [13,74], this mechanism appears not to have occurred, or was at least limited, in the cryogenic cyclic-aged PI3. This could promote the previously reported graphite dusting [18,75,76]. However, this interpretation remains an inference, supported only indirectly by the higher water loss rate of the aged specimens observed in the TGA experiment (Figure 2e), and direct verification is left to future work.

## 4. Conclusions

This study investigated the effects of cryogenic cyclic aging on the thermal, mechanical, and tribological performance of five PI-based materials, which has not been examined before. These materials included neat polyimides with different polymer structures and polyimides reinforced with MoS_2_, graphite, and/or PTFE.

Cryogenic cyclic aging had minimal effects on the weight and thermal stability of polyimide-based materials, although it significantly altered the viscoelastic behaviour, increasing the creep up to 57% and the residual strain by a maximum of 94%, depending on the material composition. Cryogenic cyclic aging induced shrinkage in the polymer matrix, with only partial recovery to its original state after aging cycles. Thermal stresses at the filler/matrix interface may lead to microcrack formation. Microstructural changes did not lead to a decrease in macroscopic mechanical properties, likely due to the length of the cyclic aging period.

The effects of cryogenic cyclic aging on fracture toughness were material-dependent. After 12 cryogenic cycles, a statistically significant increase in the *K*_1*c*_ value was confirmed only for PI2, with an average increase of up to 93%. The apparent increases observed for PI1 (40%) and PI3 (24%) were not statistically significant and are therefore reported as trends, while PI4 and PI5 showed no prominent change. The quasi-ductile fracture mode persisted after cryogenic cyclic aging. Repeated cycles of matrix shrinkage and relaxation led to the accumulation of thermal stress and delamination in the fillers or at the filler/matrix interface.

The tribological performance of the cyclic-aged materials varied depending on the polymer structure and material composition. In air, the most pronounced changes were observed for PI3 and PI5, for which the wear rate decreased significantly after aging. In particular, PI3 and PI5 showed a 26% and 41% reduction in wear rate after 12 cycles, which was associated with changes in transfer film formation. In contrast, the changes in the coefficient of friction and wear rate for PI1, PI2, and PI4 were less pronounced or not statistically significant under the selected testing conditions.

In vacuum, the effects of cryogenic cyclic aging were more severe and clearly different from the behaviour observed in air. The most critical result was obtained for the graphite-filled PI3, with the wear rate increased by 10,920% after 12 cycles. Cryogenic cyclic aging influenced the energy dissipation mechanisms, molecular chain mobility, and the accumulation of residual stresses, which together led to changes in transfer film formation and wear mechanisms.

Overall, minor changes in mechanical and tribological behaviour were already observed after four cryogenic aging cycles, but these changes became more evident with increasing numbers of cycles. In the present study, twelve cycles corresponded to approximately twelve weeks of cyclic aging. Longer aging times may further amplify the observed changes and could reveal more pronounced deterioration or stabilisation trends depending on the polymer structure and filler composition.

## Figures and Tables

**Table 1:** Compositions of the PI-based materials in this study.

Commercial Material	MoS2	Graphite	PTFE	Polymer Structure
PI1	-	-	-	1
PI2	x	-	-	
PI3	-	x	-	
PI4	-	x	x	
PI5	-	-	-	2

**Table 2:** The parameters of the tribological test.

Environment	Tribometer	Load, N	Speed, m/s	Distance, km
Air (10^5^ Pa)	CETR UMT-2 (Bruker, Billerica, MA, USA)	2	0.1	6.5
Vacuum (10^−^^5^ Pa)	RTEC MVT-2 (RTEC instruments, San Jose, CA, USA)			

**Table 3:** ANOVA results for the effects of cryogenic cyclic aging on the normalised weight of PI-based materials.

Material	F	*p*
PI1	472.2155	4.98 × 10^−9^
PI2	338.5718	1.86 × 10^−8^
PI3	21.64034	5.92 × 10^−4^
PI4	265.2907	4.87 × 10^−8^
PI5	22.20879	5.43 × 10^−4^

**Table 4:** ANOVA results for the effects of cryogenic cyclic aging on the fracture toughness of PI-based materials. Significant differences are highlighted in bold.

Material	F	*p*
PI1	1.985	0.253
PI2	10.786	**0.038**
PI3	0.406	0.693
PI4	0.041	0.959
PI5	0.692	0.556

**Table 5:** ANOVA results for the effects of cryogenic cyclic aging on CoF and wear rate of PI-based materials tested in air. Significant differences are highlighted in bold.

	CoF		Wear Rate	
Material	F	*p*	F	*p*
PI1	3.357	0.090	1.881	0.265
PI2	0.482	0.707	5.587	0.058
PI3	13.619	**0.007**	39.734	**0.003**
PI4	2.789	0.129	1.993	0.252
PI5	1.541	0.300	757.227	**9.82 × 10^−9^**

## Data Availability

The original contributions presented in this study are included in the article. Further inquiries can be directed to the corresponding authors.

## Abbreviations

The following abbreviations are used in this manuscript:

ANOVA	Analysis of variance
ASTM	American Society for Testing and Materials
CoF	Coefficient of friction
CTE	Coefficient of thermal expansion
DMA	Dynamic mechanical analysis
DSC	Differential scanning calorimetry
MoS2	Molybdenum disulfide
ODA	4,4′-Diaminodiphenyl ether
PEEK	Polyetheretherketone
PI	Polyimide
PMDA	Pyromellitic dianhydride
PTFE	Polytetrafluoroethylene
SEM	Scanning electron microscopy
TGA	Thermogravimetric analysis

## Appendix A

**Table A1:** Pairwise Welch’s *t*-test results with Holm correction for CoF values of PI-based materials showing a significant aging effect in ANOVA.

Material	Comparison	*p*
**PI3**	0 cycles vs. 4 cycles	0.098
	0 cycles vs. 8 cycles	0.065
	0 cycles vs. 12 cycles	1
	4 cycles vs. 8 cycles	1
	4 cycles vs. 12 cycles	0.561
	8 cycles vs. 12 cycles	0.561
